# Supplementary material for: Single-cell transcriptomics reveals FXR1 as an actionable target for siRNA therapy in ovarian cancer
Source: Nat Commun. 2026 Apr 3;17:4803. doi: 10.1038/s41467-026-71468-y (PMC13219712; doi:10.1038/s41467-026-71468-y)

Supplementary Figure 1

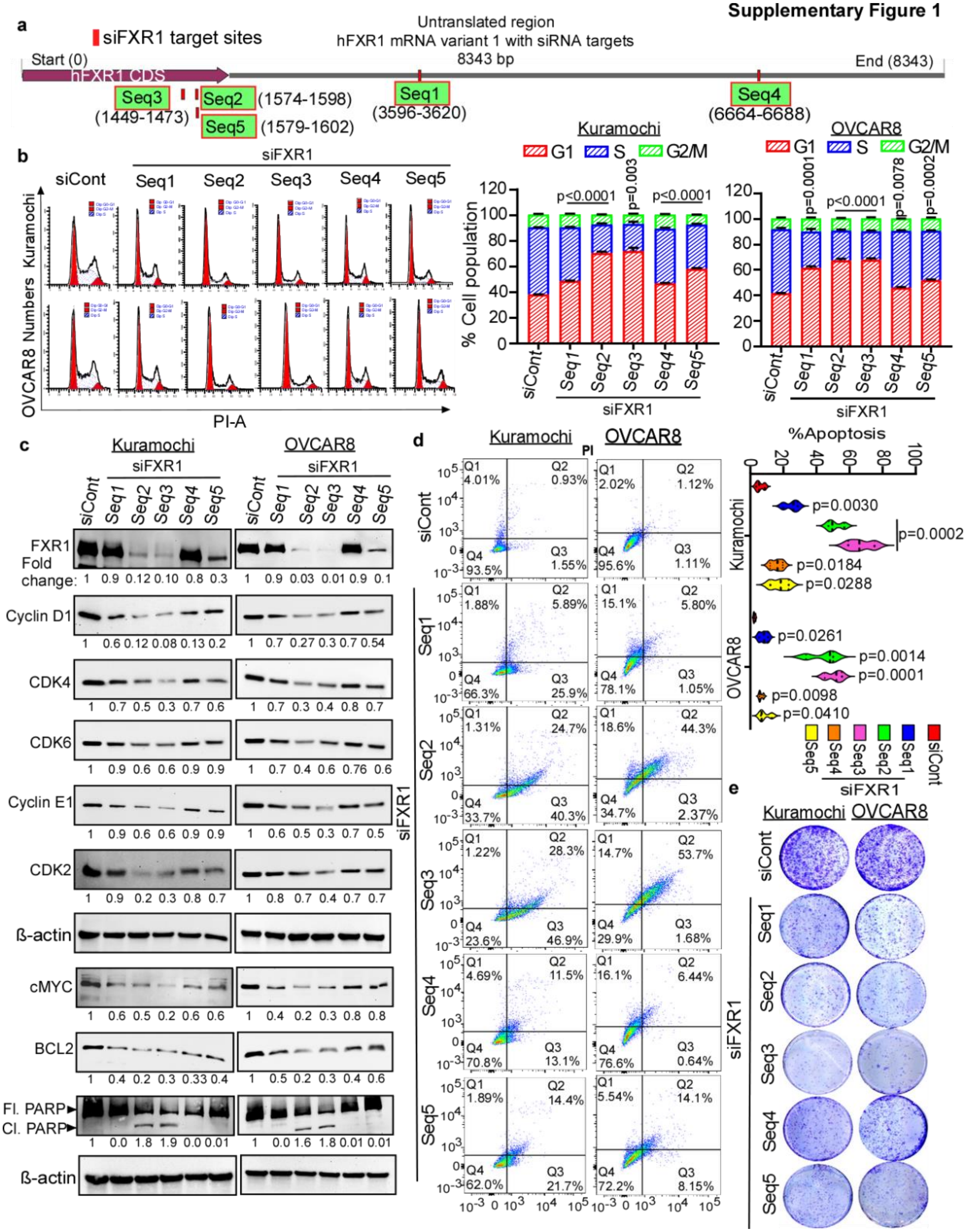

Supplementary Fig.1. FXR1 knockdown using target specific FXR1 siRNAs promotes cell death in ovarian cancer cells. a, Schematic map shows complementary binding position of FXR1-specific siRNAs within the CDS and the UTR of human FXR1 mRNA variant 1. **b,** Representative histograms show flow cytometry based cell cycle analysis of ovarian cancer cells were transfected with 2.5 nM siCont or FXR1 siRNAs (seq1-5) for 48 hr (left). Quantitative graphs represent the percentages of cell populations in G1, S, and G2/M phases of the cells (right) (n = 3, represents three biologically independent samples). Error bars indicate mean ± SEM. Significance was determined by unpaired two-tailed Student's t-test. **c,** Western blots show the levels of indicated proteins after 48 hr of transfection (n=3, represents three biologically independent samples). β-actin, loading control. Fold change in protein levels were quantitated with respect to the control and presented below each blot. **d**, Representative dot plots show flow cytometry analysis of annexin V-FITC and PI-stained cells were transfected with 2.5 nM siCont or FXR1 siRNAs (seq1-5) after 48 hr of transfection. Q2 and Q3 populations represent late and early apoptotic cells respectively and Q4 represents live cells (left). Quantitative graph to show %apoptosis in ovarian cancer cells (right) (n = 3, represents three biologically independent samples). Error bars indicate mean ± SEM. Significance was determined by unpaired two-tailed Student's t-test. **e,** Representative images of ovarian cancer cells colonies were transfected with 2.5 nM siCont or FXR1 siRNAs (seq1-5) for 48 hr (n=3, represents three biologically independent samples).

Supplementary Figure 2

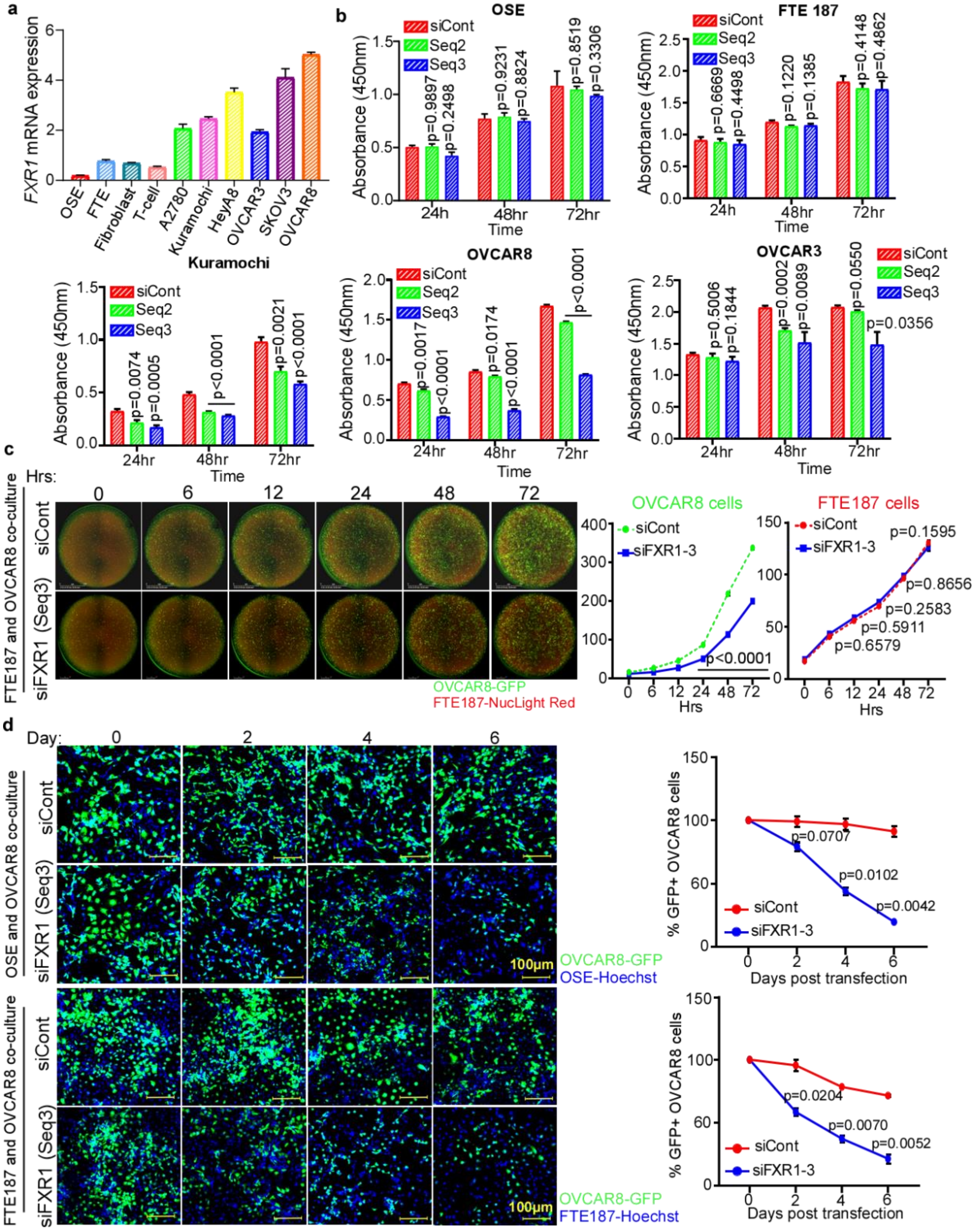

Supplementary Fig. 2. FXR1 siRNAs are not cytotoxic to normal cells. a, Quantitative bar graphs show *FXR1* mRNA expression in normal human OSE, ovarian surface epithelial cells, FTE, fallopian tube epithelial cells, fibroblast, PBMC T-cells and human ovarian cancer cell lines. *β-actin;* loading control (n=3, represents three biologically independent samples). **b,** OSE, FTE, Kuramochi, OVCAR8 and OVCAR3 cells were transfected with two individual FXR1 siRNAs (seq2 or seq3), at 2.5 nM concentration up to 72 hr. The number of viable cells were quantitated after transfection using CCK8 assay (n=5, represents five biologically independent samples). Bars represent a mean normalized absorbance at 450 nm. Error bars indicate mean ± SEM. Significance was determined by unpaired two-tailed Student's t-test. **c,** Representative images show GFP+ OVCAR8 cells co-cultured with NucLight Red-labeled FTE cells for 24 hr, followed by treatment with siFXR1 (seq3) or siCont at 2.5 nM. Images were captured every 6 hr over 3 days using the IncuCyte live-cell analysis system (left). Scale bars represent 2 mm.The graph displays time-dependent cell count curves for GFP+ OVCAR8 cells and NucLight Red-labeled FTE cells were treated with siCont or FXR1 siRNAs (right) (n=3, represents three biologically independent samples). Error bars indicate mean ± SEM. Significance was determined by unpaired two-tailed Student's t-test. **d,** Representative fluorescence images captured from four random fields of GFP+ OVCAR8 cells co-cultured with OSE and FTE cells for 24 hr, followed by transfection with 2.5 nM of FXR1 siRNAs (seq3) or siCont (left), respectively. Cell numbers were quantified at different timepoints using live cell imaging with GFP and Hoechst stain. Proportion of OVCAR8 cells were quantitated by dividing the number of GFP+ cells by the total number of Hoechst+ nuclei (n=3, represents three biologically independent samples) (right). Scale bars represent 100 µm.

Error bars indicate mean ± SEM. Significance was determined by unpaired two-tailed Student's t-test.

Supplementary Figure 3

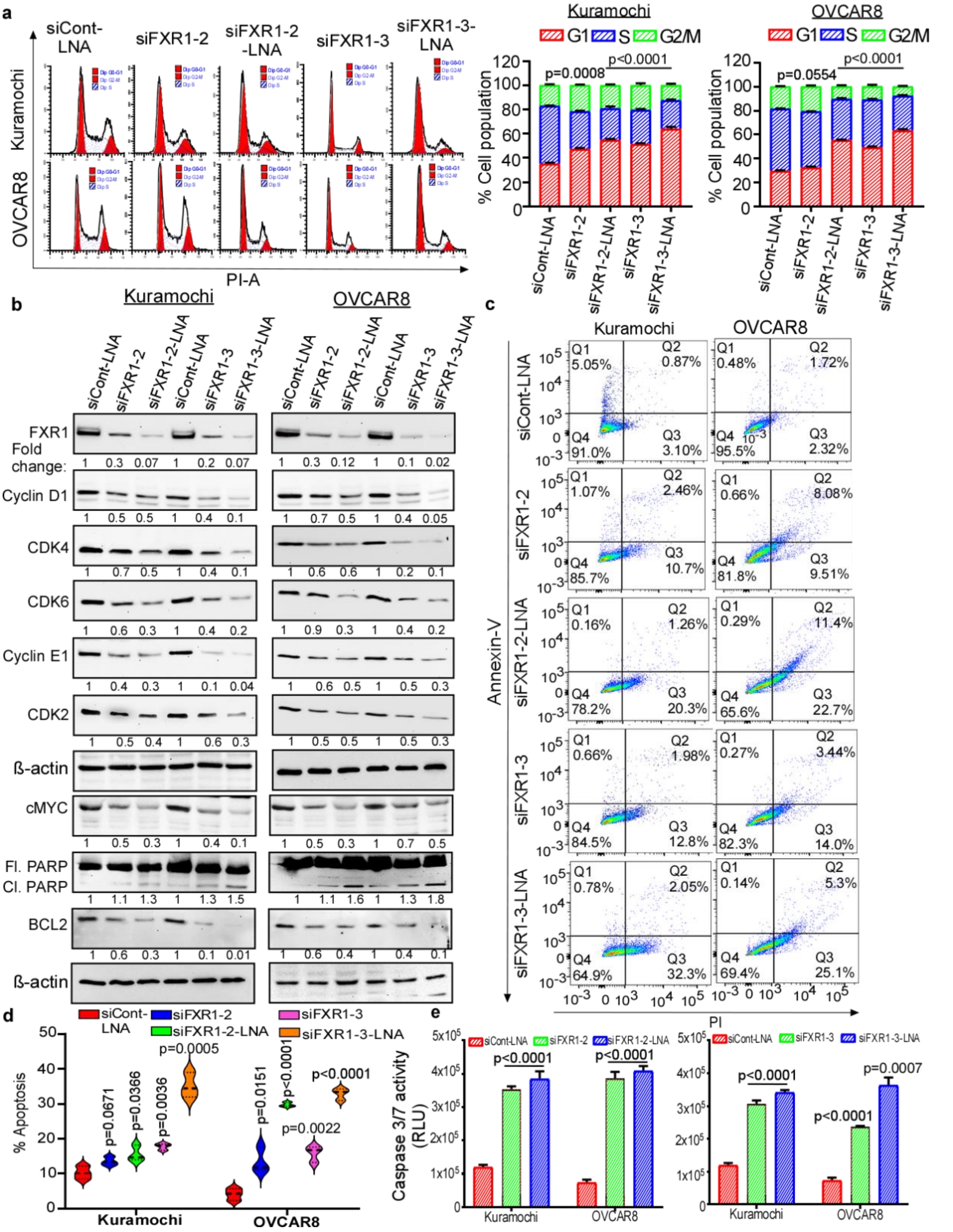

Supplementary Fig. 3. LNA modified FXR1 siRNAs induce anti-tumor effects in ovarian cancer cells. a, Representative histograms show flow cytometry analysis of cell cycle performed for ovarian cancer cells were transfected with 0.5 nM siCont-LNA, native siFXR1-2, siFXR1-2-LNA, native siFXR1-3 or siFXR1-3-LNA (right). Graphs represent the percentages of cell populations in G1, S, and G2/M phases of the cells (right) (n=3, represents three biologically independent samples). Error bars indicate mean ± SEM. Significance was determined by unpaired two-tailed Student's t-test. **b,** Western blots show the levels of indicated proteins identified after 48 hr of transfection indicated siRNA. β-actin, loading control. Fold change in protein levels were quantitated with respect to the control and presented below each blot. **c,** Representative dot plots show flow cytometry of cells were immunostained for annexin V-FITC and PI-stained cells after 48 hr of transfection of 0.5 nM concentration of siCont-LNA, native siFXR1-2, siFXR1-2-LNA, native siFXR1-3 or siFXR1-3-LNA . **d,** Quantitative graph shows % apoptosis of ovarian cancer cells in **c** (n=3, represents three biologically independent samples). Error bars indicate mean ± SEM. Significance was determined by unpaired two-tailed Student's t-test. **e,** Quantitative bar graph for caspase 3/7 activity recorded after 48 hr of transfection of 0.5 nM concentration of siCont-LNA, native siFXR1-2, siFXR1-2-LNA, native siFXR1-3 or siFXR1-3-LNA treatment in ovarian cancer cells (n=3, represents three biologically independent samples). Error bars indicate mean ± SEM. Significance was determined by unpaired two-tailed Student's t-test.

Supplementary Figure 4

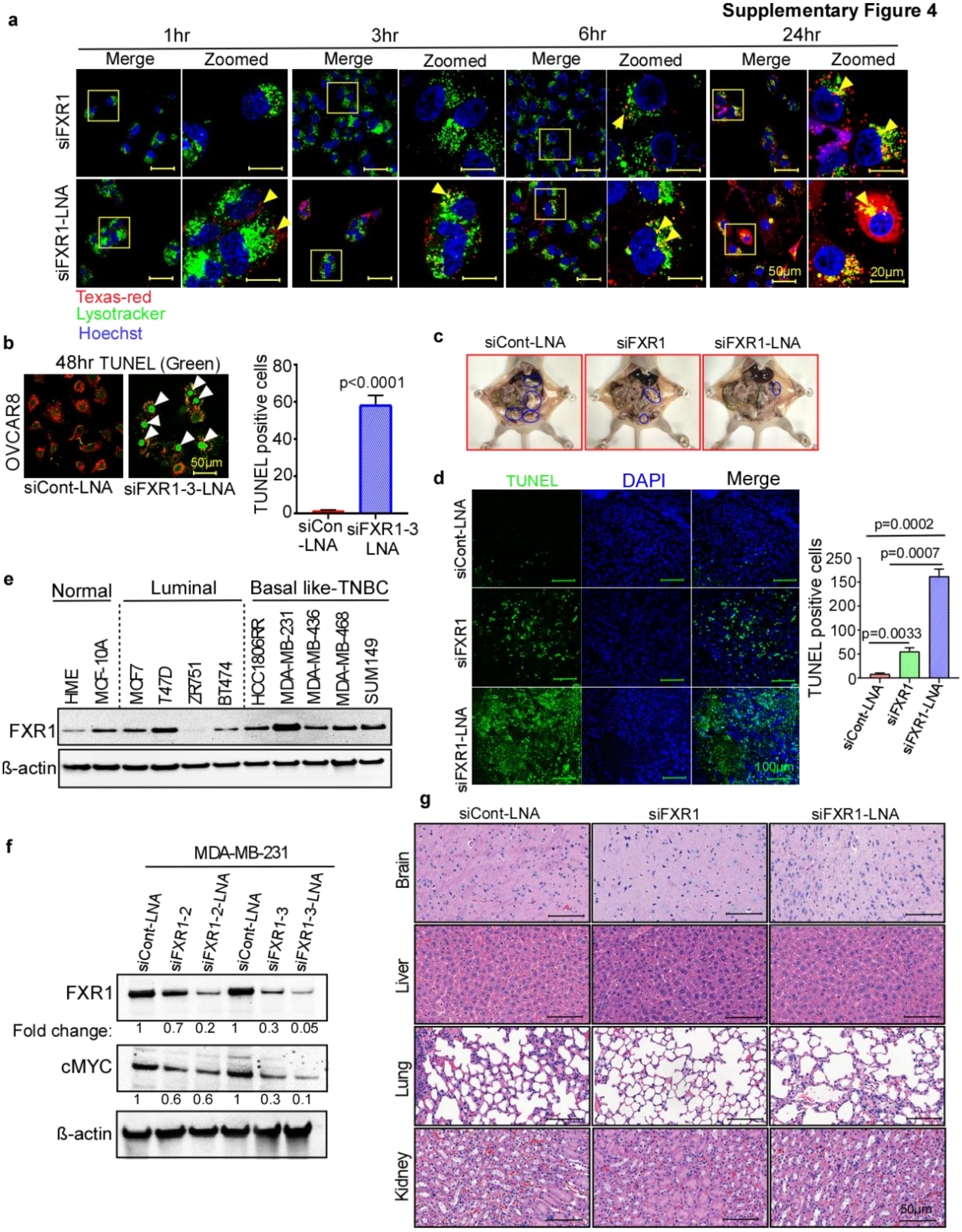

Supplementary Fig. 4. LNA modified FXR1 siRNA demonstrates improved cellular uptake and cell death consequently *in vitro* and *in vivo*. a, Representative images of intracellular distribution of siRNA were captured from five random fields of ovarian cancer cells were transfected with Texas red labelled native siFXR1 or siFXR1-LNA. The nucleus was stained with Hoechst, the lysosome vesicles were labeled by Lysotracker Red, and the siRNA were labeled with Texas red. Scale bars represent 50 μm in unzoomed images and 20 μm in zoomed images (n=3, represents three biologically independent samples). **b,** Representative images captured from five random fields of TUNEL-stained ovarian cancer cells are shown to assess the apoptotic effects of siFXR1-3-LNA 48 hr after transfection. Apoptotic cells were marked as green fluorescence after TUNEL staining observed under a fluorescent microscope (arrow indicates TUNEL-positive cells). Scale bars represent 50 μm. Bar graph shows number of apoptotic cells as determined by TUNEL assay (n=3, represents three biologically independent samples). Significance was determined by unpaired two-tailed Student's t-test. **c,** Representative image (left) of the anatomy of peritoneal cavity of mice from each group. Areas circled in blue indicate tumor nodules formed on peritoneal organs. **d,** Representative TUNEL staining (green fluorescence, left) images of the tumor sections were performed from Fig. 3a. Scale bars represent 100 μm. Bar graph shows number of apoptotic cells in tumor tissues as determined by TUNEL assay (right). A total of 3 fields from each treatment group were counted. Error bars indicate mean ± SEM. Significance was determined by unpaired two-tailed Student's t-test. (n=3, represents three biologically independent samples). **e**, Western blot analysis of FXR1 levels in human normal breast cells and cancer cells. β-actin, loading control (n=3, represents three biologically independent samples). **f**,

Western blot analysis shows FXR1 levels after native siFXR1 (seq2 or seq3) or siFXR1-LNA (seq2 or seq3) were transfected in MDA-MB-231 cells. β-actin, loading control (n=3, represents three biologically independent samples). Fold change in protein levels were quantitated with respect to the control and presented below each blot. **g**, Representative H&E images of main organs from toxicity study of the mice were treated with siCont-LNA, siFXR1 or siFXR1-LNA are presented; (n=5 mice per group). Scale bars represent 50 μm.

Supplementary Figure 5

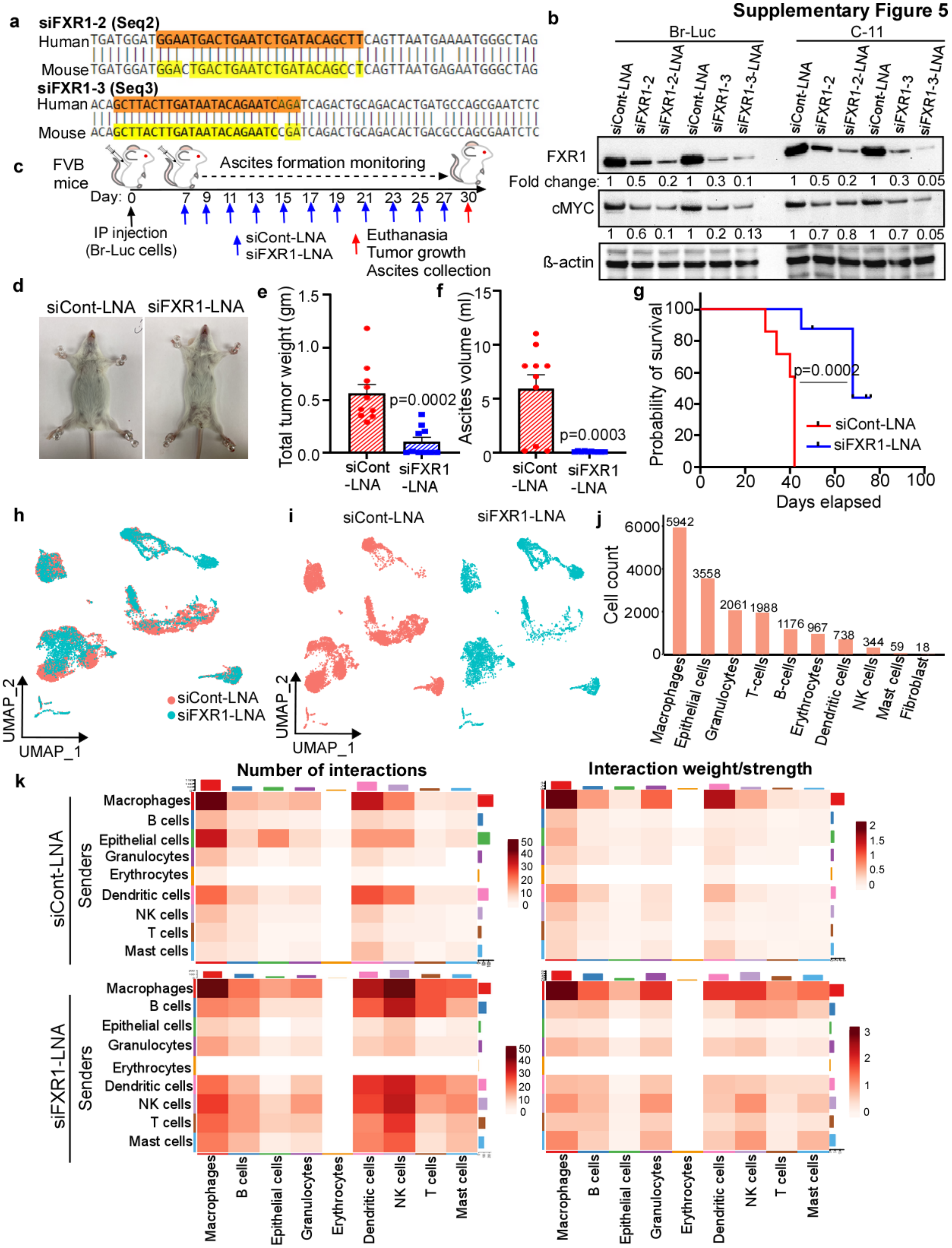

Supplementary Fig. 5. Single-cell RNA sequencing (scRNA-seq) reveals immune cell modulation supporting anti-tumor mechanisms following FXR1 RNAi in immunocompetent mice. a, Sequence complementarity between human and mouse siRNA sequences. **b,** Immunoblots show FXR1 levels after native siFXR1 (seq2 or seq3) or siFXR1-LNA (seq2 or seq3) were transfected in mouse ovarian cancer cell lines. β-actin, loading control. Fold change in protein levels were quantitated with respect to the control and presented below each blot. (n=3, represents three biologically independent samples). **c**, Schema shows the overall study design of scRNA-seq analysis using ascites samples collected from PEI encapsulated siCont-LNA or siFXR1-LNA treated FVB/N mice (n=10 mice per group). On day 30, all mice were euthanized, ascites fluid and tumors were collected for further analysis. **d,** Representative images of mice show ascites fluid formation. Primary and disseminated tumors were collected from c, then **e,** total tumor weight and **f,** ascitic fluid volume were recorded. Error bars indicate mean ± SEM. Significance was determined by unpaired two-tailed Student's t-test. **g,** Kaplan-Meier survival plot of immunocompetent FVB/N mice were injected with Br-Luc mouse ovarian cancer cells and then treated with PEI-carried siCont-LNA (n=10 mice per group) or siFXR1-LNA (n=10 mice per group). Significance was determined by Log-rank (Mantel-Cox) test. **h,** UMAP visualization of integrated single cells data at 0.1 resolution from scRNA-seq analysis of tumor ascites samples isolated from siCont-LNA or siFXR1-LNA treated mice. The combination of two biological replicates for each population was presented for each group. **i,** UMAP plot shows abundance of different cell types in the TME of siCont-LNA or siFXR1-LNA treated ascitic samples. **j,** Bar graph illustrates the cell count in each cluster. **k,** Heatmaps shows the quantity and strength of interaction

between indicated cell populations in siCont-LNA or siFXR1-LNA treated group. Mice drawings in panel (c) were created by Canvas X Pro.

Supplementary Figure 6

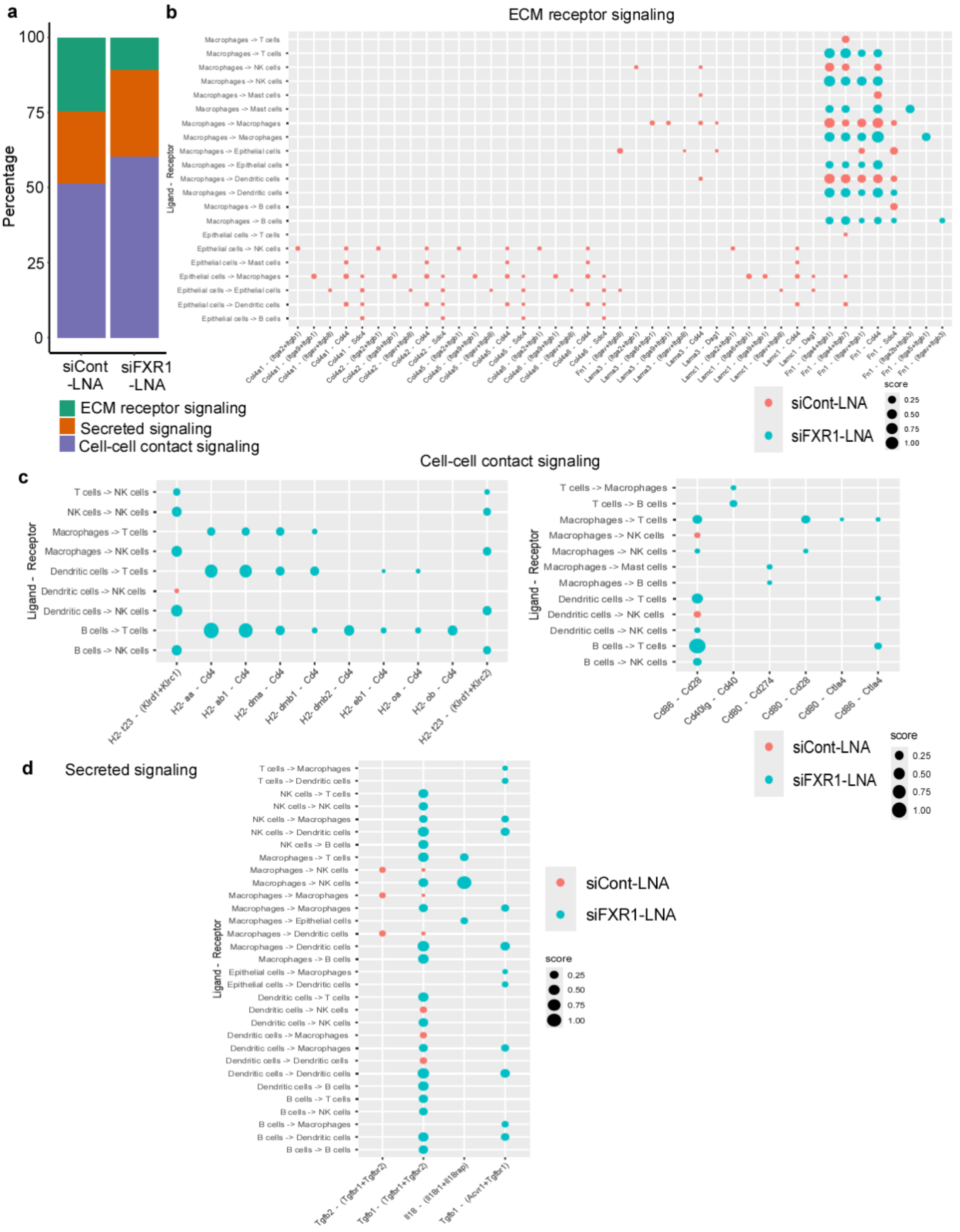

Supplementary Fig. 6. CellChat analysis reveals reduced ECM-receptor signaling and increased secreted and cell-cell contact signaling upon FXR1 silencing. a, Stacked bar graph shows change in percentage of genes associated with the indicated pathways among cell populations isolated from siCont-LNA or siFXR1-LNA treated groups. **b**, **c,** and **d,** Dot plot shows the top ligand–receptor interactions contributing to the ECM-receptor signaling, and cell-cell contact and secreted signaling among epithelial cells, macrophages, T cells, B cells, NK cells and DCs in siCont-LNA or siFXR1-LNA treated groups. The colors represent respective groups and the dot size represents the probability of level of communication.

Supplementary Figure 7

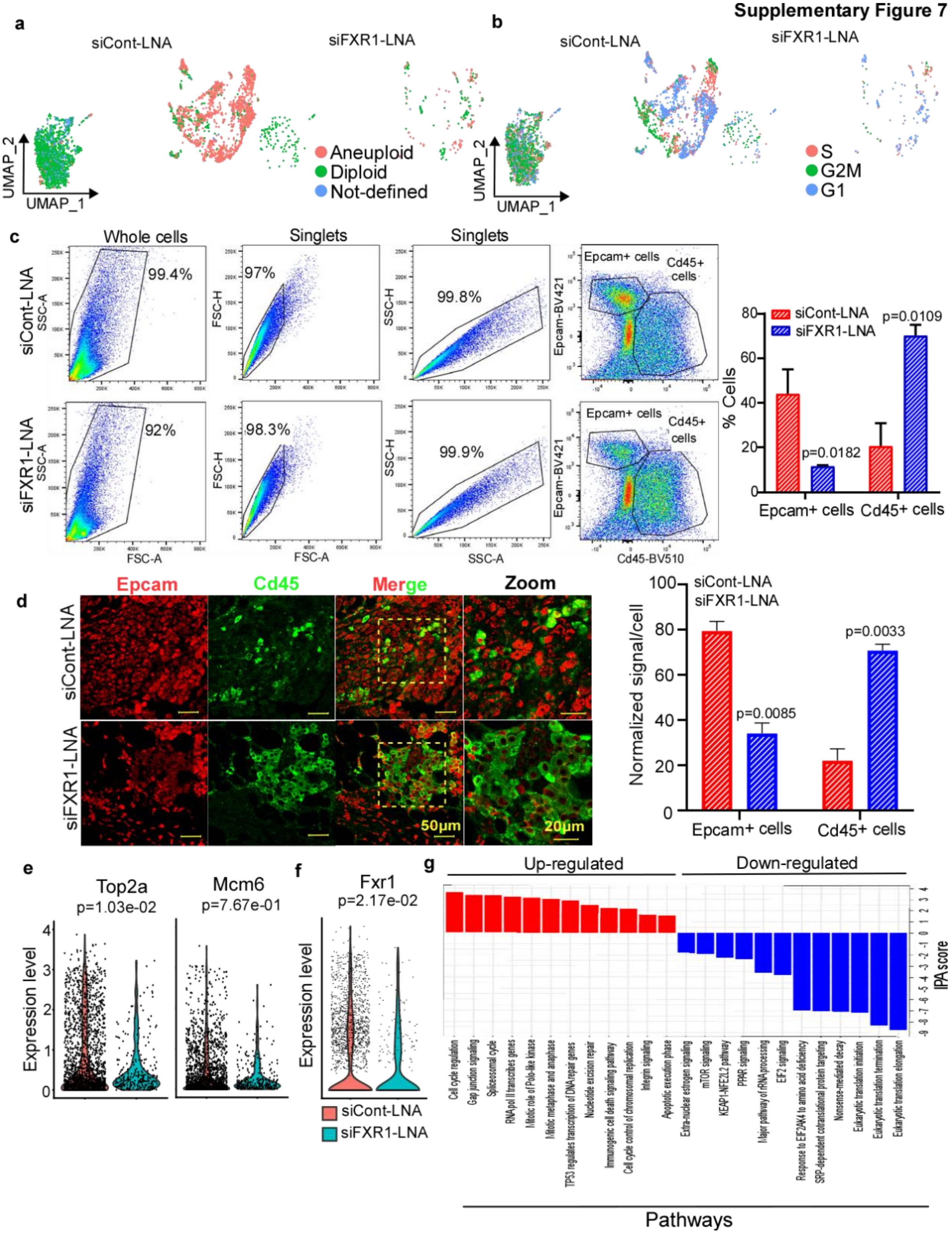

Supplementary Fig. 7. LNA modified siFXR1 reduces the levels of FXR1 in tumor cells *in vivo*. a, UMAP plots shows the chromosomal copy number variation (CNV) aneuploid, or diploid status in epithelial cells from the siCont-LNA or siFXR1-LNA groups. **b**, UMAP plots shows the distribution of cell cycle phases in epithelial cells from the siCont-LNA and siFXR1-LNA groups. **c,** Representative images show flow cytometry analysis of ascites fluid collected from control siRNA or FXR1 siRNA treated mice performed after immunostaining using $Epcam^+$ and $Cd45^+$ specific antibodies. Marked area shows $Epcam^+$ or $Cd45^+$ (left). Quantitative bar graph shows percentage of $Epcam^+$ and $Cd45^+$ cells in the samples were collected from siCont-LNA or siFXR1-LNA treated group (right) (n=3, represents three biologically independent samples). Error bars indicate mean ± SEM. Significance was determined by unpaired two-tailed Student's t-test. **d**, Representative immunofluorescence (IF) images show Epcam and Cd45 expressing cells on tumor tissue sections prepared from siCont-LNA or siFXR1-LNA treated group. Scale bars represent 50 μm and 20 μm (left). Bar graph shows quantitaion of $Epcam^+$ and $Cd45^+$ cells based on staining intensity (right) (n=3, represents three biologically independent samples). Error bars indicate mean ± SEM. Significance was determined by unpaired two-tailed Student's t-test. **e,f,** Violin plot shows the expression of genes indicated in epithelial cells subclusters in siCont-LNA or siFXR1-LNA treated groups, p-values were calculated by two-tailed Wilcoxon Rank Sum test. **g**, Bar graph shows enrichment of canonical pathways of DEGs in epithelial cells. Pathway activity scores were calculated by IPA software.

Supplementary Figure 8

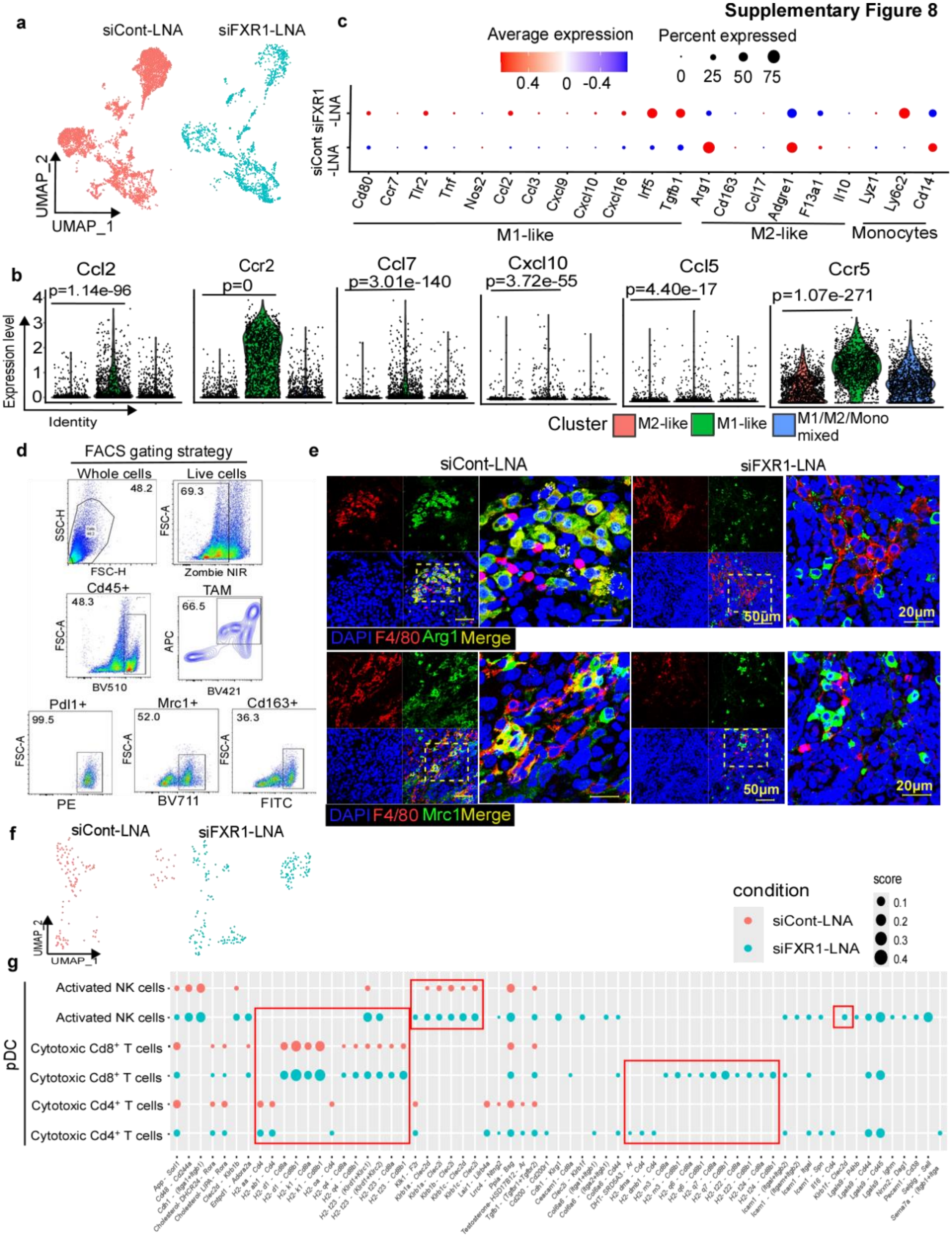

Supplementary Fig. 8. FXR1 RNAi promotes anti-tumor immune cells at the tumor site. a, UMAP plot shows macrophage subclusters in siCont-LNA and siFXR1-LNA treated groups. **b**, Violin plot shows the distribution of indicated chemokines and their receptors within macrophages subclusters, p-values were calculated by two-sided Wilcoxon rank sum test. **c,** Dot plot shows macrophage subtypes marker genes in siCont-LNA or siFXR1-LNA treated groups. **d,** Flow cytometry gating strategy used for analyzing Pdl1, Mrc1, and Cd163 expression in tumor-associated macrophages (TAMs) in mouse ascites samples. **e,** Representative immunofluorescence (IF) images captured from five random fields for F4/80, Arg1 and Mrc1 expressing cells in siCont-LNA and siFXR1-LNA treated tumor tissue sections collected from Supplementary Fig. 5c. Scale bars represent 50 μm and 20 μm (n=3, represents three tissue samples collected from three mice respectively) **f,** UMAP plot shows abundance of DCs in siCont-LNA or siFXR1-LNA treated groups. **g**, Dot plot shows the level of cell–cell communication signal initiated from pDC to activate NK cells, $Cd8^+$ and $Cd4^+$ cytotoxic T cell populations.

Supplementary Figure 9

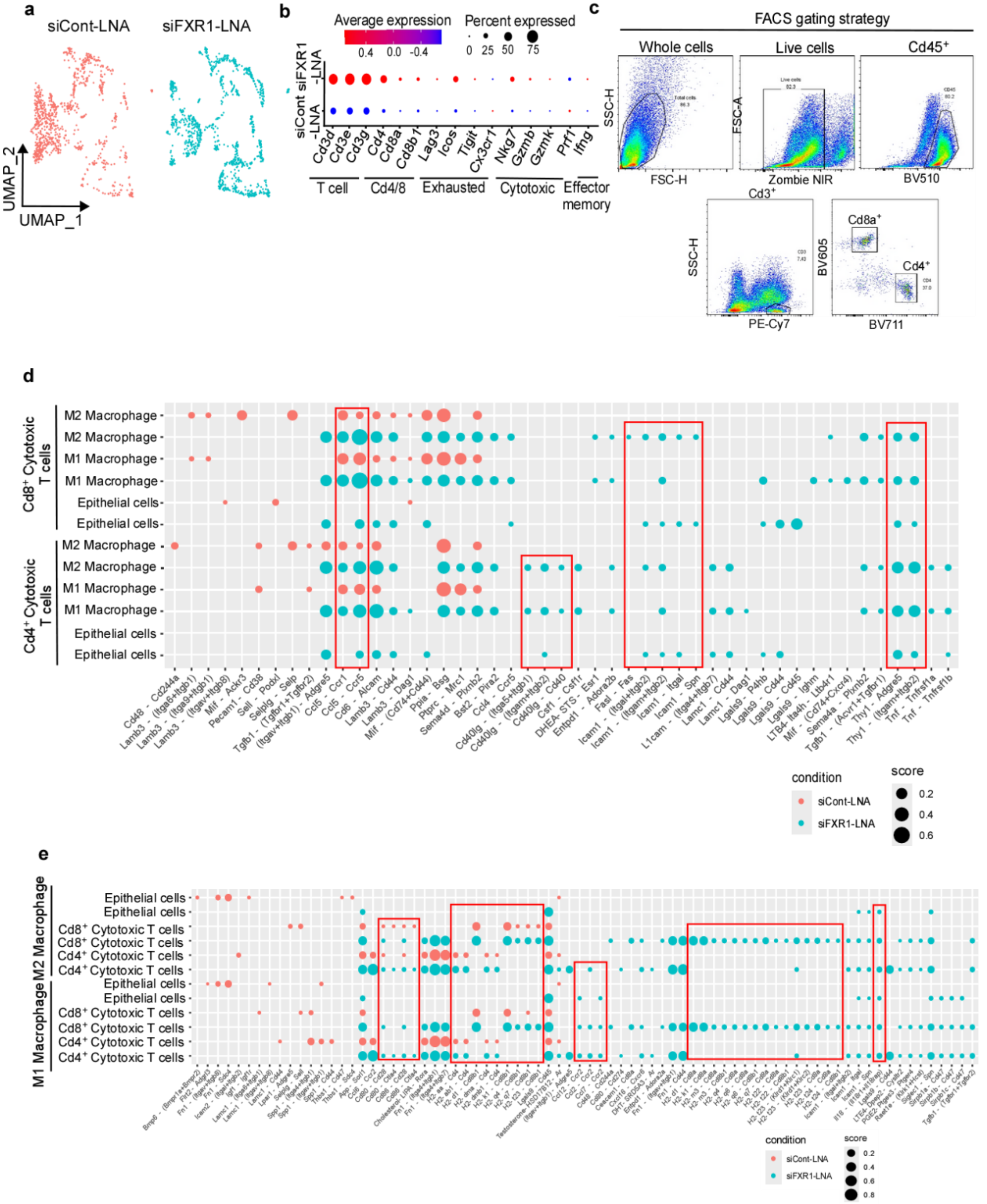

Supplementary Fig. 9. FXR1 RNAi enhances cytotoxic T cells enrichment and promotes their anti-tumor interactions with macrophages and tumor cells. a, UMAP plot shows T cell population in siCont-LNA and siFXR1-LNA groups. **b**, Dot plot shows proportions of T cell subtypes based on the markers in the siCont-LNA and siFXR1-LNA groups. **c**, Gating strategy employed for flow cytometry analyses to determine $Cd4^+$ and $Cd8^+$ T cells isolated from mouse ascites samples. **d**, Dot plot denotes the levels of cell-to-cell communication initiated from $Cd8^+$ and $Cd4^+$ T cell subtypes interact with macrophages subtypes and epithelial cells in siCont-LNA and siFXR1-LNA treated groups. **e**, Dot plot denotes the levels of cell-to-cell communication initiated from macrophages subtypes interact with $Cd8^+$ and $Cd4^+$ cytotoxic T cell subtypes in siCont-LNA and siFXR1-LNA treated groups.

Table S1: List of siRNA sequences used in this study.

siRNA	Sequence (5'-3')
Seq1	Sense: AAGAUAUCAAGUAAUUUCAUGUCCU<br>Antisense: AGGACAUGAAAUUACUUGAUAUCUU
Seq2	Sense: GGAAUGACUGAAUCUGAUACAGCUU<br>Antisense: AAGCUGUAUCAGAUUCAGUCAUUCC
Seq3	Sense: GCUUACUUGAUAAUACAGAAUCAGA<br>Antisense: UCUGAUUCUGUAUUAUCAAGUAAGC
Seq4	Sense: ACAAGCUUCUGAACUGCAAAACCUU<br>Antisense: AAGGUUUUGCAGUUCAGAAGCUUGU
Seq5	Sense: GACUGAAUCUGAUACAGCUUCAGUU<br>Antisense: AACUGAAGCUGUAUCAGAUUCAGUC
Scrambled Control siRNA	Sense: GCCAAAUUCAUGCUUGGAAUCACUC<br>Antisense: GAGUGAUUCCAAGCAUGAAUUUGGC

Table S2: Acute toxicity of siRNAs in nude mice. Athymic nude mice were injected with 10 µg/mouse of siCont-LNA, native siFXR1, or siFXR1-LNA, (n=5 mice per group) encapsulated in PEI nanoparticle (twice weekly for 4 weeks) IP. After the final IP injection, blood was collected, then serum prepared for biochemical analysis of indicated markers.

	Liver functions			Kidney functions		
**Parameters**	**ALP (U/L)**	**AST (U/L)**	**ALT (U/L)**	**TBIL (mg/dL)**	**Albumin (g/dL)**	**TP (g/dL)**
Normal Range	52-560	26-120	50-96	0.0-0.9	2.5-4.0	4.3-6.4
siCont-LNA	63.75	48.75	27.25	0.2	3.0	5.1
siFXR1	69.75	45.5	20.0	0.17	2.8	4.65
siFXR1-LNA	64.25	53.75	24.75	0.2	3.2	5.42

Aspartate Aminotransferase (AST); Alanine Aminotransferase (ALT); Alkaline Phosphatase (ALP); Total Bilirubin (TBIL); Total Protein (TP)

Supplementary Fig 1c: Western blot analysis of whole-cell lysates showing G1-phase protein levels following transfection with siFXR1 (Seq #1–5) in ovarian cancer cells.

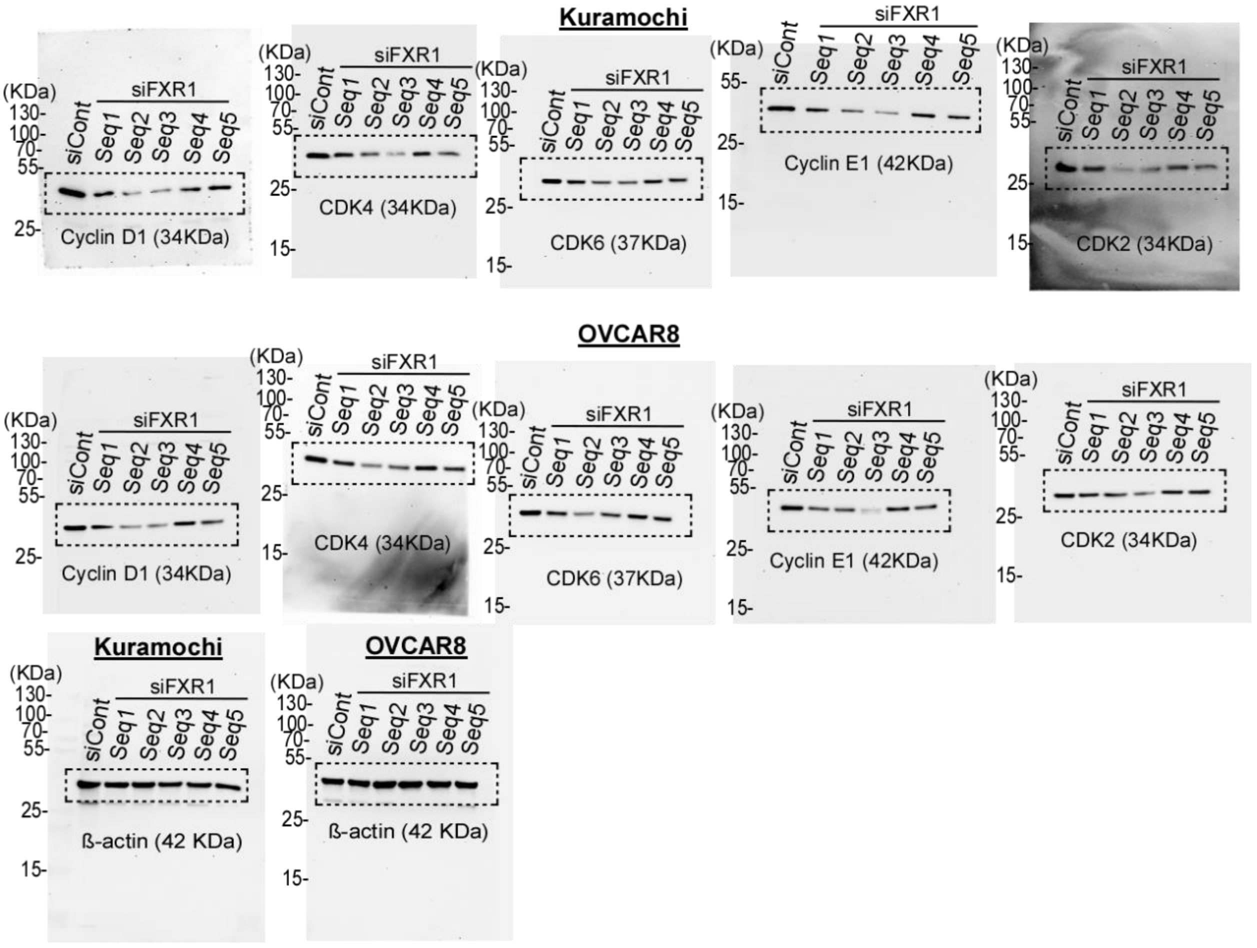

Supplementary Fig. 1c Detection of apoptosis and FXR1 target proteins level after siFXR1 (#seq1-5) transfection in ovarian cancer cells

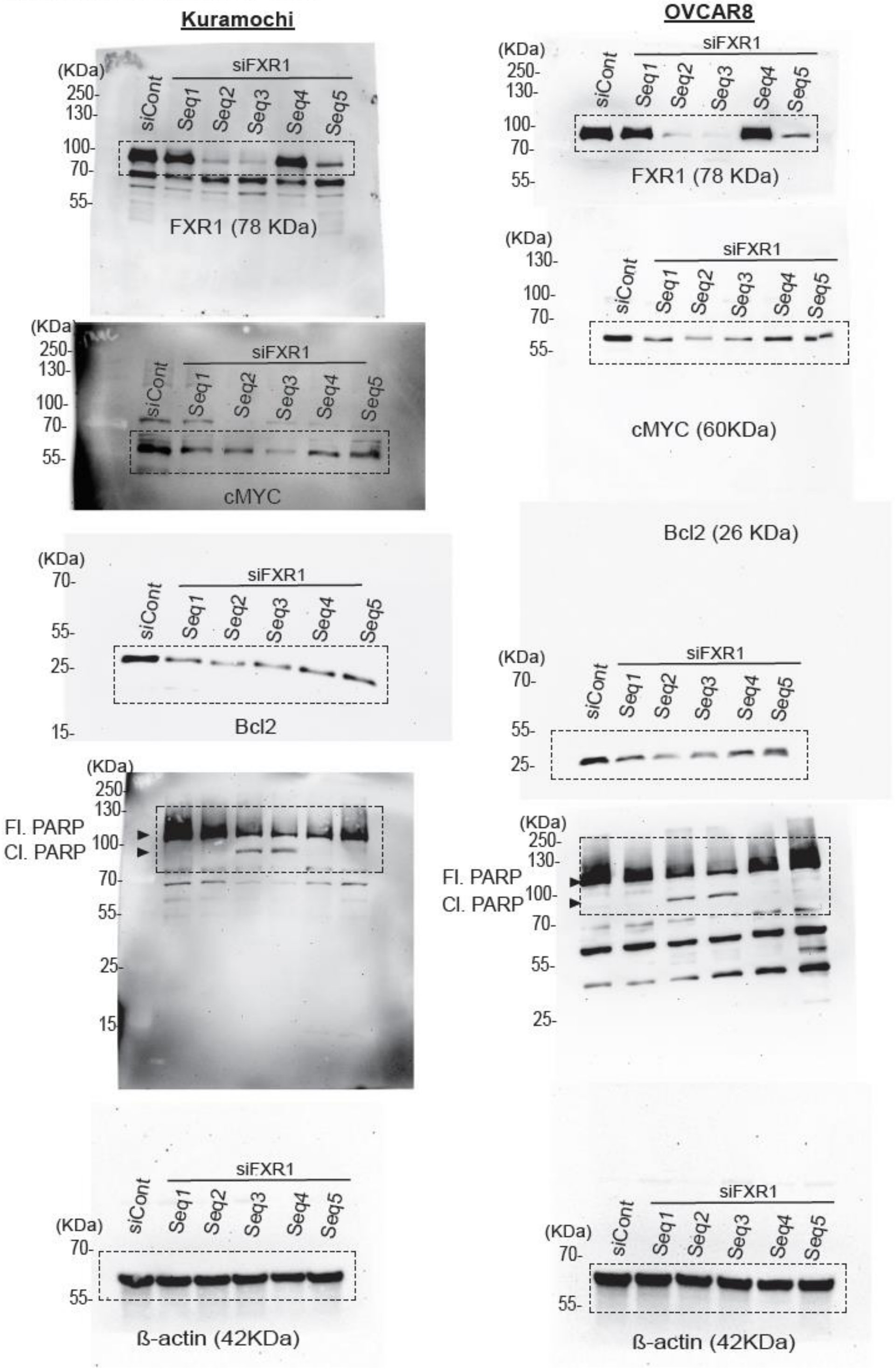

Supplementary Fig 3b: Western blot analysis of whole-cell lysates showing G1-phase protein levels following transfection with native siFXR1 and siFXR1-LNA in ovarian cancer cells.

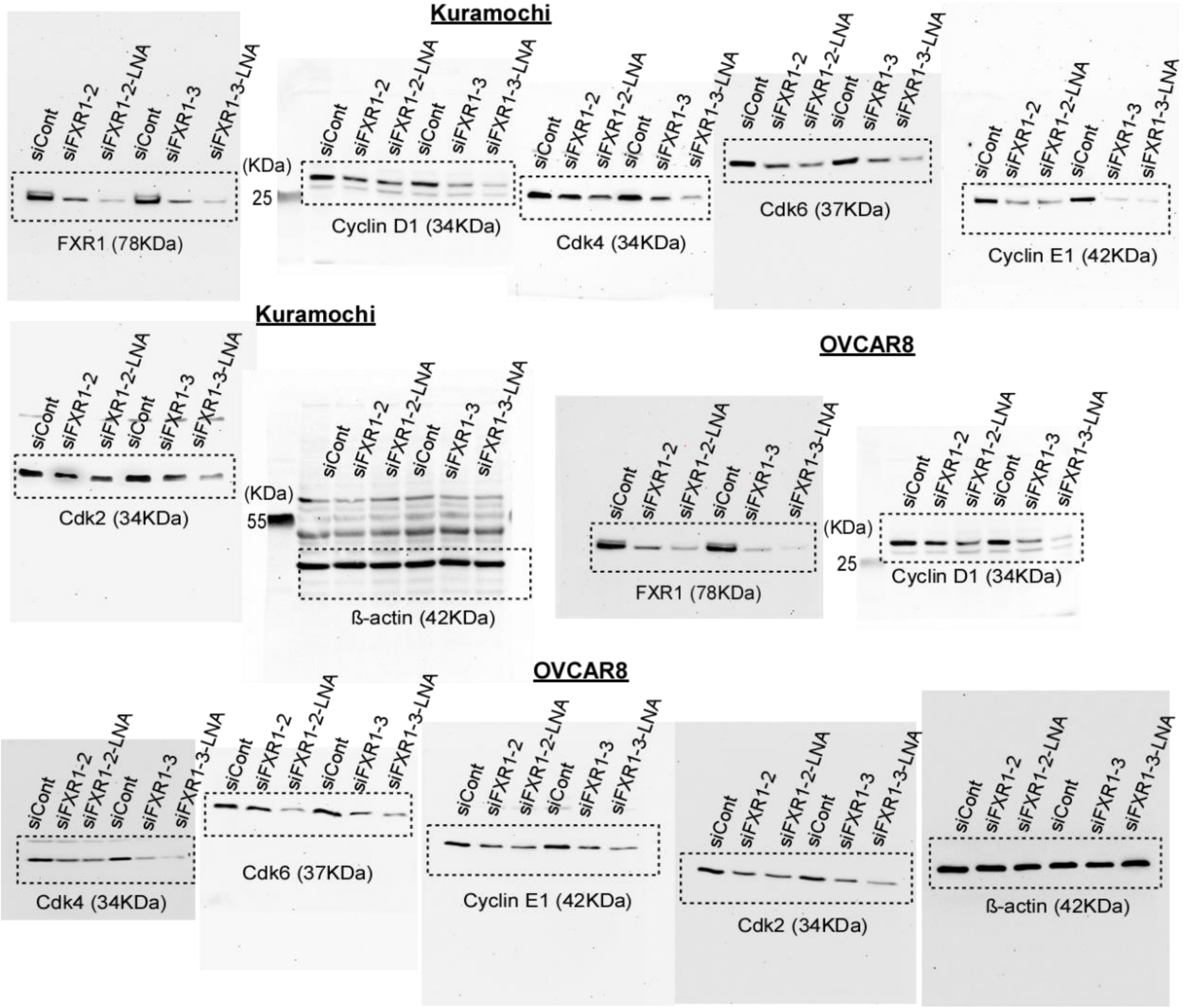

Supplementary Fig 3b: Western blot analysis of whole-cell lysates showing apoptosis-related protein levels after native siFXR1 and siFXR1-LNA transfection in ovarian cancer cells.

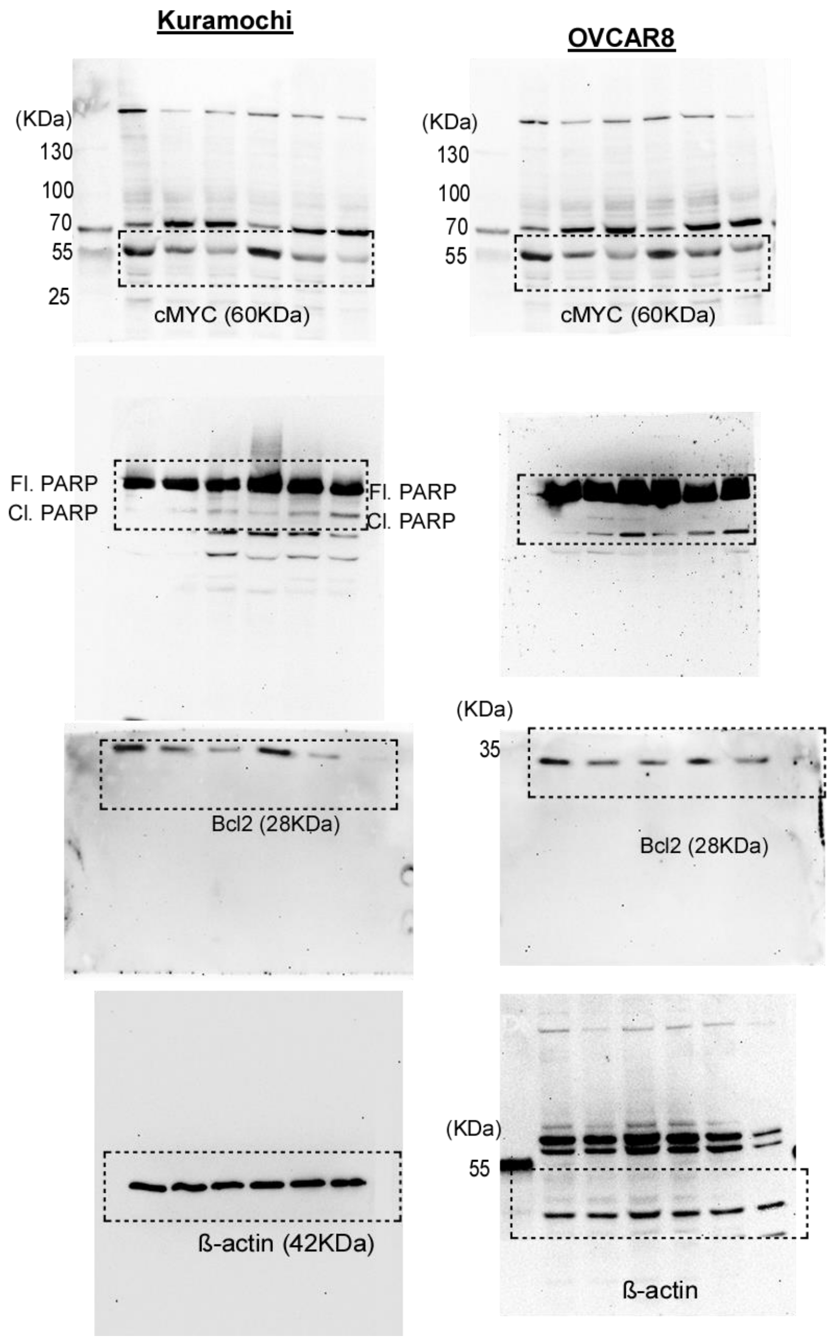

Supplementary Fig 4d: Western blots of FXR1 levels in whole cell lysate of human normal breast cells and cancer cells.

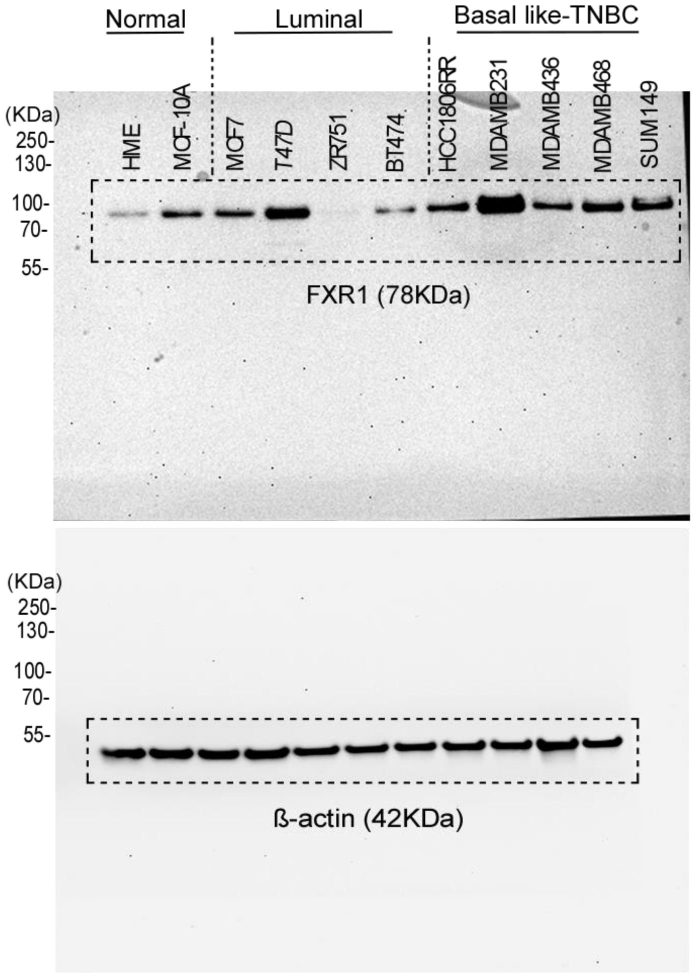

Supplementary Fig 4e: Western blots of FXR1 and cMYC levels in whole cell lysate of MDA-MB-231 cells transfected with native siFXR1 (Seq2 and Seq3) and siFXR1-LNA (Seq2 and Seq3).

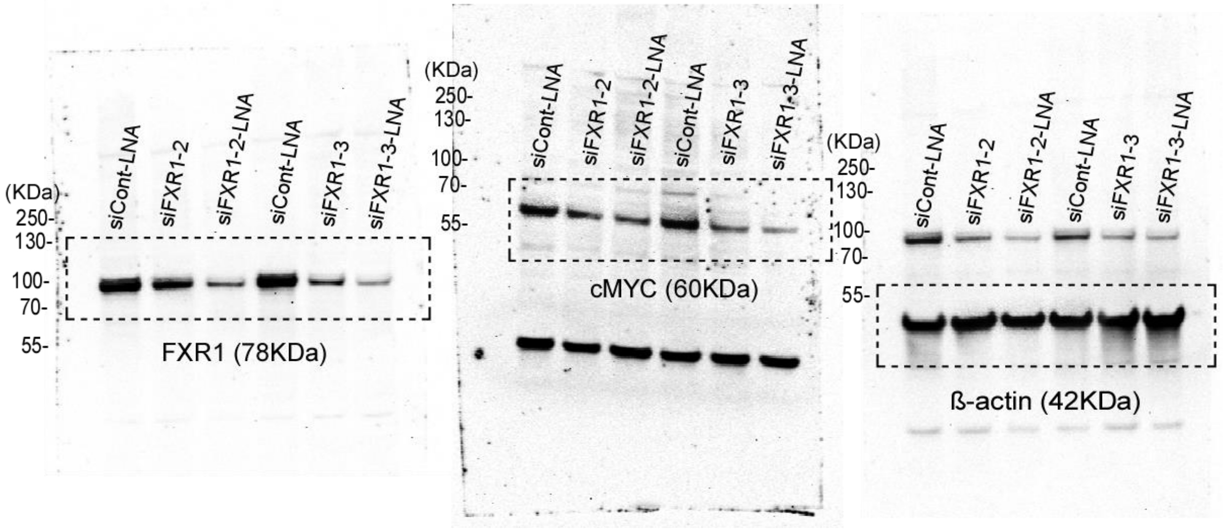

Supplementary Fig 5b: Western blots showing FXR1 and c-MYC levels in whole-cell lysates of mouse ovarian cancer cells after transfection with native siFXR1(Seq2 and Seq3) and siFXR1-LNA (Seq2 and Seq3).

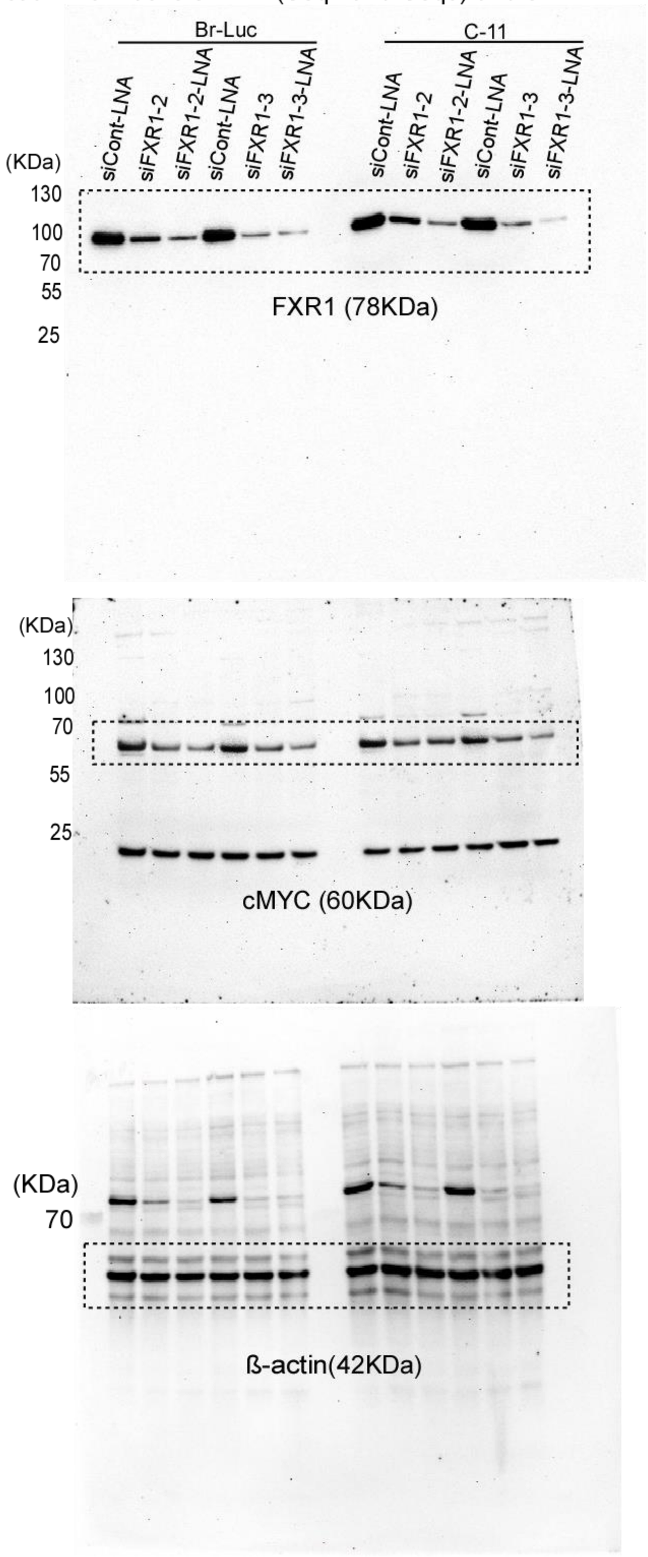

Supplement: Supplementary file 1 — Supplementary Information [file 41467_2026_71468_MOESM1_ESM.pdf]
