# Supplementary material for: Single-cell transcriptomics reveals FXR1 as an actionable target for siRNA therapy in ovarian cancer
Source: Nat Commun. 2026 Apr 3;17:4803. doi: 10.1038/s41467-026-71468-y (PMC13219712; doi:10.1038/s41467-026-71468-y)
Supplement: Supplementary file 3 — Description of Additional Supplementary Files [file 41467_2026_71468_MOESM3_ESM.pdf]

Description of Additional Supplementary Files

Supplementary Movie 1: Movie shows the level of uptake of fluorescently labeled native siFXR1 in tumor spheroids.

Supplementary Movie 2: Movie shows the level of uptake of fluorescently labeled siFXR1-LNA in tumor spheroids.

Supplementary Data 1: Table shows differentially expressed genes in epithelial cell clusters used for (Ingenuity Pathway Analysis) IPA. p-values were calculated by two-sided Wilcoxon Rank Sum test.
