# Supplementary material for: Single-cell transcriptomics reveals FXR1 as an actionable target for siRNA therapy in ovarian cancer
Source: Nat Commun. 2026 Apr 3;17:4803. doi: 10.1038/s41467-026-71468-y (PMC13219712; doi:10.1038/s41467-026-71468-y)
Supplement: Supplementary file 7 — Reporting Summary [file 41467_2026_71468_MOESM7_ESM.pdf]

nature portfolio

Corresponding author(s): *Double-anonymous peer review submissions: write DAPR and your manuscript number here instead of author names.*

Last updated by author(s): Feb 18, 2026

Reporting Summary

Nature Portfolio wishes to improve the reproducibility of the work that we publish. This form provides structure for consistency and transparency in reporting. For further information on Nature Portfolio policies, see our Editorial Policies and the Editorial Policy Checklist.

Statistics

For all statistical analyses, confirm that the following items are present in the figure legend, table legend, main text, or Methods section.

n/a	Confirmed	
☐	☒	The exact sample size (*n*) for each experimental group/condition, given as a discrete number and unit of measurement
☐	☒	A statement on whether measurements were taken from distinct samples or whether the same sample was measured repeatedly
☐	☒	The statistical test(s) used AND whether they are one- or two-sided<br>*Only common tests should be described solely by name; describe more complex techniques in the Methods section.*
☒	☐	A description of all covariates tested
☒	☐	A description of any assumptions or corrections, such as tests of normality and adjustment for multiple comparisons
☐	☒	A full description of the statistical parameters including central tendency (e.g. means) or other basic estimates (e.g. regression coefficient) AND variation (e.g. standard deviation) or associated estimates of uncertainty (e.g. confidence intervals)
☐	☒	For null hypothesis testing, the test statistic (e.g. *F*, *t*, *r*) with confidence intervals, effect sizes, degrees of freedom and *P* value noted<br>*Give P values as exact values whenever suitable.*
☒	☐	For Bayesian analysis, information on the choice of priors and Markov chain Monte Carlo settings
☒	☐	For hierarchical and complex designs, identification of the appropriate level for tests and full reporting of outcomes
☒	☐	Estimates of effect sizes (e.g. Cohen's *d*, Pearson's *r*), indicating how they were calculated

Our web collection on statistics for biologists contains articles on many of the points above.

Software and code

Policy information about availability of computer code

Data collection	Single cell RNA-sequencing data was generated using NovaSeq X Plus (PE150-10B) platform.
Data analysis	Aim 4.2 software LSM 510; ImageJ software; IncuCyte® S3 software; OpenComet (version 1.3.1) software; Living Image software; Case Viewer software; IPA software; CellRanger (version 7.0.0);R packages used include: CopyKAT (version 1.1.0); CellChat (version 2.1.2); ggplot2 (version 3.5.1); Graphpad Prism 7; ModFit LT software; FlowJo 10 (version 10.9.0); Seurat (version 5.1.0); Monocle 3 (version 3.0)

For manuscripts utilizing custom algorithms or software that are central to the research but not yet described in published literature, software must be made available to editors and reviewers. We strongly encourage code deposition in a community repository (e.g. GitHub). See the Nature Portfolio guidelines for submitting code & software for further information.

Data

Policy information about availability of data

All manuscripts must include a data availability statement. This statement should provide the following information, where applicable:

- Accession codes, unique identifiers, or web links for publicly available datasets
- A description of any restrictions on data availability
- For clinical datasets or third party data, please ensure that the statement adheres to our policy

All raw scRNA-seq data generated for this study has been deposited in the NCBI Gene Expression Omnibus (GEO) portal with the accession number GSE292799.

Research involving human participants, their data, or biological material

Policy information about studies with human participants or human data. See also policy information about sex, gender (identity/presentation), and sexual orientation and race, ethnicity and racism.

Reporting on sex and gender	Sex and gender information was not relevant to the study where PBMC samples were used. Therefore such information was not taken under consideration.
Reporting on race, ethnicity, or other socially relevant groupings	The research was not involved in race, ethnicity, or other social relevant groupings. Therefore, such information were not taken under consideration.
Population characteristics	Individuals whose blood samples were collected were not reported for cancer incidence.
Recruitment	Blood samples were collected from Froedtert Hospital and Medical College of Wiscsonsin Tissue Bank in de-identified manner.
Ethics oversight	Blood samples from human were collected for PBMC isolation in de-identified manner with a written informed consent in compliance with the institutional review board approved protocol (PRO00041601) by the Ethics Committee of Froedtert Hospital and Medical College of Wisconsin. All samples were handled in compliance with the Declaration of Helsinki.

Note that full information on the approval of the study protocol must also be provided in the manuscript.

Field-specific reporting

Please select the one below that is the best fit for your research. If you are not sure, read the appropriate sections before making your selection.

☒ Life sciences ☐ Behavioural & social sciences ☐ Ecological, evolutionary & environmental sciences

For a reference copy of the document with all sections, see nature.com/documents/nr-reporting-summary-flat.pdf

Life sciences study design

All studies must disclose on these points even when the disclosure is negative.

Sample size	In vitro experiments: Sample size of at least three was used in most experiments for statistical analysis, for cell viability sample size of four to five were used.<br>For in vivo experiments:<br>n=7 mice/group were used for ovarian tumor study,<br>n=5 mice/group were used for siRNAs toxicity study,<br>n=10 mice/group were used for scRNA-seq and survival study<br>n=5 mice/group for breast tumor study
Data exclusions	For scRNA-seq data, we excluded low-quality cells if abnormalities exist in (1) the number of expressed genes; (2) the proportion of mitochondrial genes counts.
Replication	We repeated in vitro experiments at least two-three times, and confirmed reproducibility of data.
Randomization	No method of randomization was used for in vitro experiments.<br>Method of randomization was used in in vivo experiment were mice randomized and sorted based on bio-luminescence signal for further treatment purpose.
Blinding	No blinding was performed in this study.

Reporting for specific materials, systems and methods

We require information from authors about some types of materials, experimental systems and methods used in many studies. Here, indicate whether each material, system or method listed is relevant to your study. If you are not sure if a list item applies to your research, read the appropriate section before selecting a response.

Materials & experimental systems

n/a	Involved in the study
☐	☒ Antibodies
☐	☒ Eukaryotic cell lines
☒	☐ Palaeontology and archaeology
☐	☒ Animals and other organisms
☒	☐ Clinical data
☒	☐ Dual use research of concern
☒	☐ Plants

Methods

n/a	Involved in the study
☒	☐ ChIP-seq
☐	☒ Flow cytometry
☒	☐ MRI-based neuroimaging

Antibodies

Antibodies used

All antibodies were commercially purchased and included:
Alexa Fluor 568 goat anti-rabbit, Life Technologies, Cat# F2765;
Alexa Fluor goat anti-mouse 488, Life Technologies, Cat# F2761;
Anti-rabbit IgG, HRP-linked, Cell Signaling Technology, Cat# 7074P2;
Bax, Cell Signaling Technology, Cat# 2772;
Bcl2, Santa Cruz Biotechnology, Cat# sc-7382;
Cdk2, Cell Signaling Technology, Cat# 2546;
Cdk4, Cell Signaling Technology, Cat# 12790;
Cdk6, Cell Signaling Technology , Cat# 13331;
Cleaved caspase3, Cell Signaling Technology, Cat# 9669;
cMYC, Cell Signaling Technology, Cat# 5605S;
Cyclin D1, Cell Signaling Technology, Cat# 12205;
Cyclin E1, Cell Signaling Technology, Cat# 20808;
FXR1 Cell Signaling Technology Cat#12295S;
Ki-67, Cell Signaling Technology, Cat# 9027;
PARP, Cell Signaling Technology, Cat#9532;
F4/80 Cell Signaling Technology Cat#30325;
ARG1/Arginase 1, Santa Cruz Biotechnology, Cat#sc-271430;
CD206 (Mrc1), Santa Cruz Biotechnology, Cat#sc-58986
Epcam, Proteintech, Cat # 21050-1-AP
Cd45, Proteintech, Cat # 60287-1-Ig)
β-actin, Cell Signaling Technology, Cat# 4970;
Anti-mouse Cd4-BV711, Biolegend Clone: GK1.5; Cat# 100447;
Anti-mouse Cd8a-BV605, Biolegend Clone: 53-6.7; Cat# 100744;
Anti-mouse Cd45-BV510, Biolegend Clone: 30-F11; Cat# 103138;
Anti-mouse Cd3-PE/Cy7, Biolegend Cat# 100220;
Anti-mouse Cd206 (Mrc1)-BV711 Biolegend Clone: C068C2; Cat# 141727;
Anti-mouse Pdl1-PE, Biolegend Clone: 10F.9G2; Cat# 124308;
Anti-mouse F4/80-APC ThermoFischer Sceintific,Clone: BM8; Cat# 17-4801-82;
Anti-mouse Cd163-FITC, ThermoFischer Sceintific, Cat# 11-1631-82;
Anti mouse Cd16/32, Biolegend, Cat# 156603;
Anti-mouse Cd326 (EpCAM), Biolegend, Cat# 118225;
Anti mouse Cd11b-BV605, Biolegend, Cat# 101237

Validation

All the antibodies used in this study were commercial antibodies and validated by the companies.

Eukaryotic cell lines

Policy information about cell lines and Sex and Gender in Research

Cell line source(s)

Cell line details are provided in Methods section of the manuscript.

Authentication

Authenticity of the cell lines used were confirmed by STR characterization at IDEXX Bioanalytics Services (Columbia, MO)

Mycoplasma contamination

Cells were routinely tested and deemed free of PlasmotestTM Mycoplasma Detection Kit (InvivoGen, San Diego, CA).

Commonly misidentified lines
(See ICLAC register)

N/A

Animals and other research organisms

Policy information about studies involving animals; ARRIVE guidelines recommended for reporting animal research, and Sex and Gender in Research

Laboratory animals

Athymic nude mice (J:NU, Strain #:007850), and FVB/NJ mice (Strain #001800) approximately 4 to 6 weeks old female were

Laboratory animals	purchased from Jackson Laboratories (Bar Harbor, ME, USA).
Wild animals	No
Reporting on sex	Female
Field-collected samples	No
Ethics oversight	All mice were housed under specific pathogen-free conditions in accordance with guidelines and therapeutic interventions approved by the Institutional Animal Care and Use Committee (IACUC) at the Medical College of Wisconsin.

Note that full information on the approval of the study protocol must also be provided in the manuscript.

Plants

Seed stocks	*Report on the source of all seed stocks or other plant material used. If applicable, state the seed stock centre and catalogue number. If plant specimens were collected from the field, describe the collection location, date and sampling procedures.*
Novel plant genotypes	*Describe the methods by which all novel plant genotypes were produced. This includes those generated by transgenic approaches, gene editing, chemical/radiation-based mutagenesis and hybridization. For transgenic lines, describe the transformation method, the number of independent lines analyzed and the generation upon which experiments were performed. For gene-edited lines, describe the editor used, the endogenous sequence targeted for editing, the targeting guide RNA sequence (if applicable) and how the editor was applied.*
Authentication	*Describe any authentication procedures for each seed stock used or novel genotype generated. Describe any experiments used to assess the effect of a mutation and, where applicable, how potential secondary effects (e.g. second site T-DNA insertions, mosiacism, off-target gene editing) were examined.*

Flow Cytometry

Plots

Confirm that:

- [ ] The axis labels state the marker and fluorochrome used (e.g. CD4-FITC).
- [ ] The axis scales are clearly visible. Include numbers along axes only for bottom left plot of group (a 'group' is an analysis of identical markers).
- [ ] All plots are contour plots with outliers or pseudocolor plots.
- [ ] A numerical value for number of cells or percentage (with statistics) is provided.

Methodology

Sample preparation	Ascites sample preparation for Flow cytometry: Ascites collected from euthanized mice were centrifuged at 2000 rpm. ACL lysis buffer was added according to the volume of the pellet (10 ml of buffer for 2 ml of pellet) and incubated for 5 min to lyse red blood cells. 1X PBS was added, and the preparation was centrifuged again to compact the cell pellet. The mixture was filtered through a 70 µm filter to collect tumor cells and TILs. These cells were then washed and counted, and 2x106 cells were collected to be stained for flow cytometry.
Instrument	Data was collected on a BD Bioscience LSRII, BD LSRFortessa X-20 and BDSymphony A5 SE.
Software	The data was collected using FACSDiva version 8 software. Data was analyzed using ModFit LT software and FlowJo version 10.9.0.
Cell population abundance	Cell population abundance is shown in Supplementary Figures.
Gating strategy	Gating strategy is given in Supplementary Figures.

- [x] Tick this box to confirm that a figure exemplifying the gating strategy is provided in the Supplementary Information.
