# Supplementary material for: Single-cell transcriptomics reveals FXR1 as an actionable target for siRNA therapy in ovarian cancer
Source: Nat Commun. 2026 Apr 3;17:4803. doi: 10.1038/s41467-026-71468-y (PMC13219712; doi:10.1038/s41467-026-71468-y)

Figure 1a: Western blots for FXR1 level after siFXR1 (#seq1-5) transfection in ovarian cancer whole cell lysates.

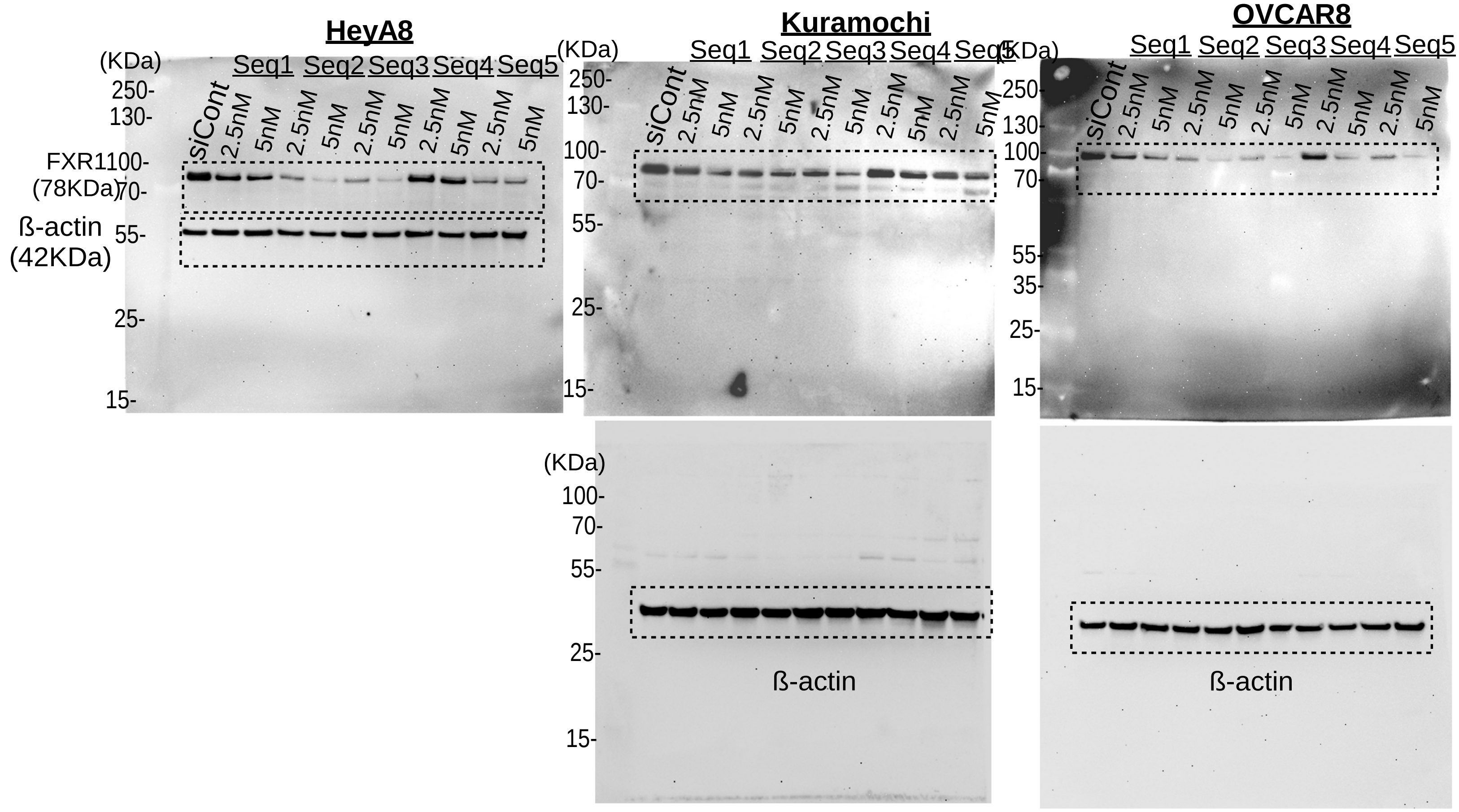

Figure 1e: Western blots for FXR1 level after native siFXR1 and siFXR1-LNA transfection in Kuramochi whole cell lysate.

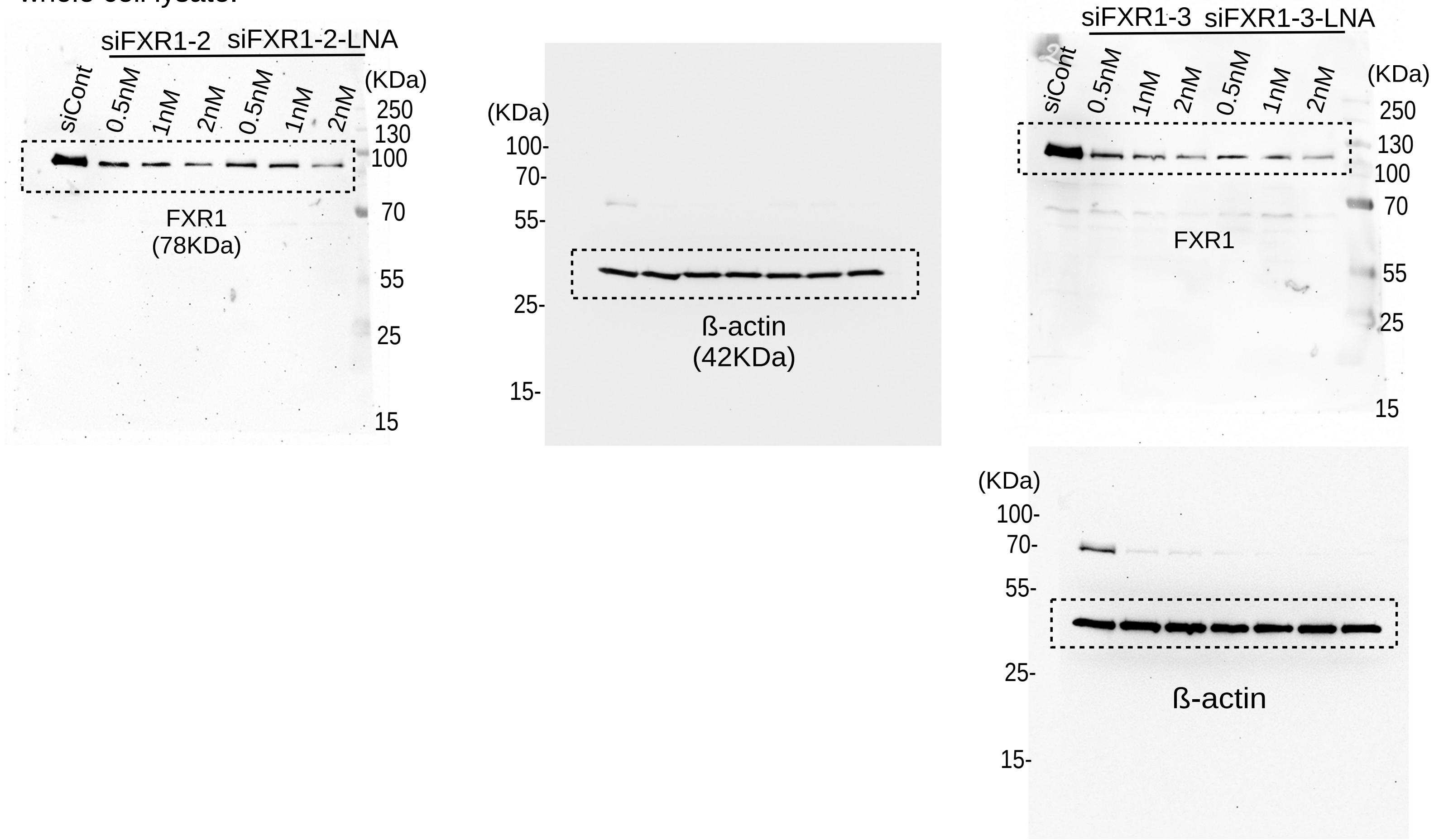

Figure 2e: RNA gel for stability of native siFXR1, siFXR1-LNA and their JetPEI nanocomplex.

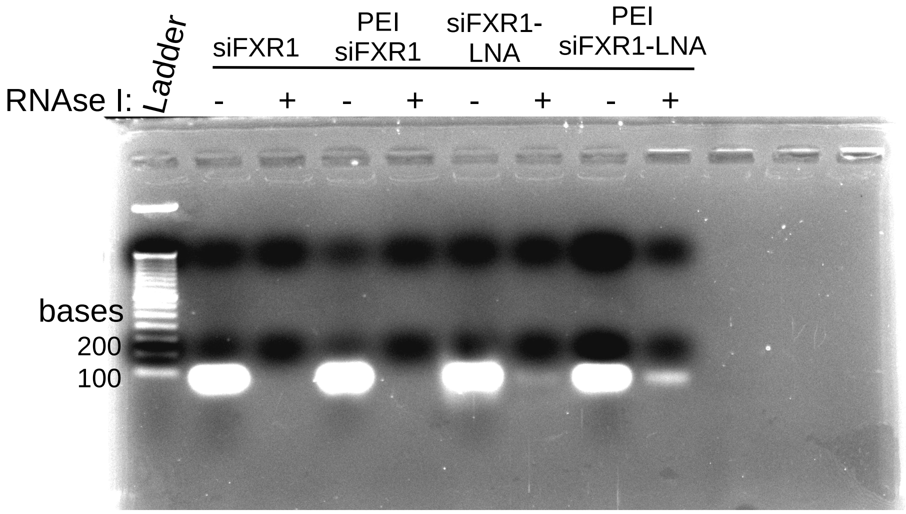

Figure 2f: PAGE images for native siFXR1 and FXR1-LNA nanocomplex incubated in 10% human serum (HS) and mouse serum (MS) at 37ºC for the time points indicated.

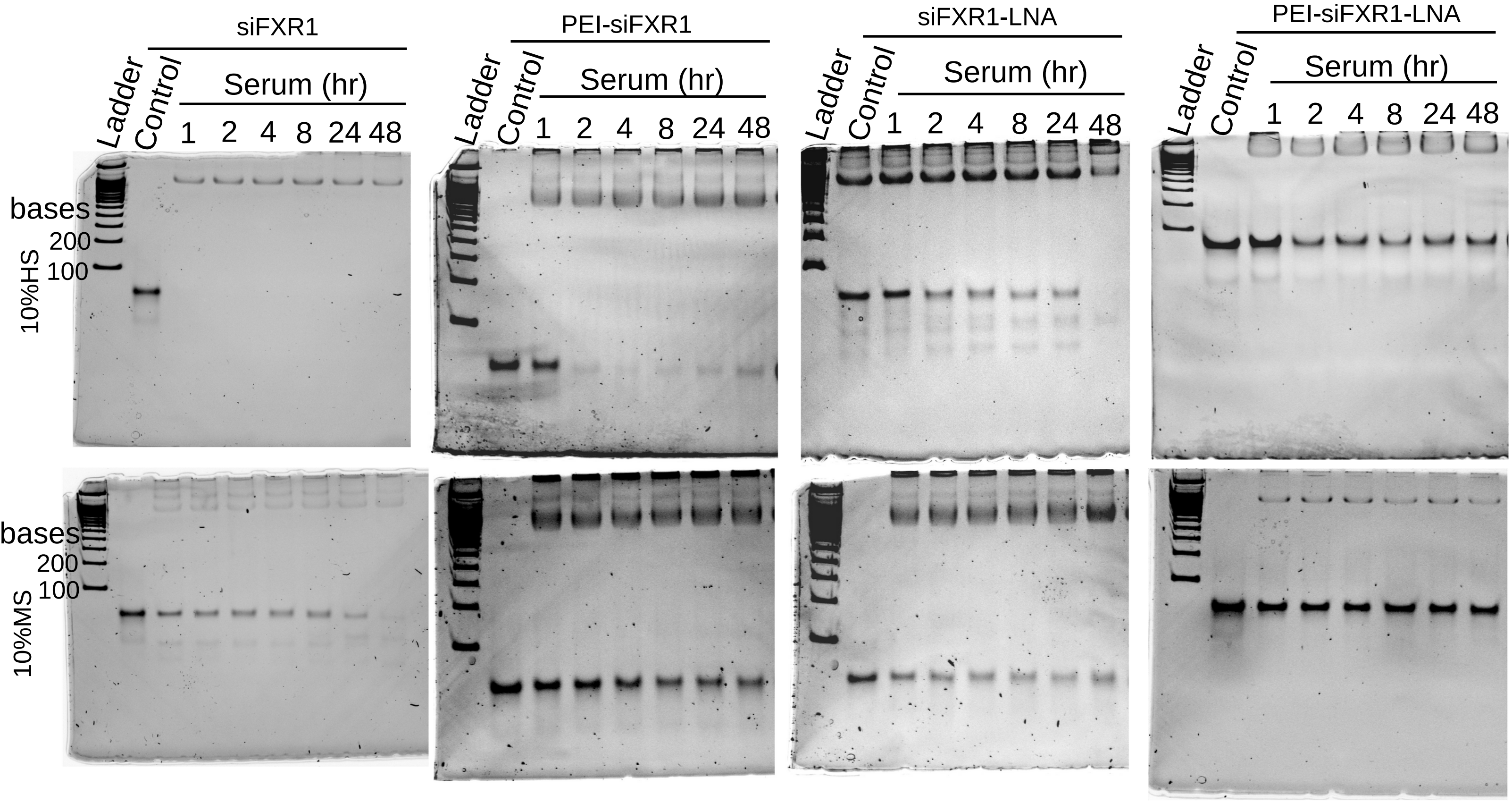

Figure 3e: Western blots showing FXR1 and c-MYC levels in whole tumor lysates from the OVCAR8 in vivo model following treatment with native siFXR1 or siFXR1-LNA.
.

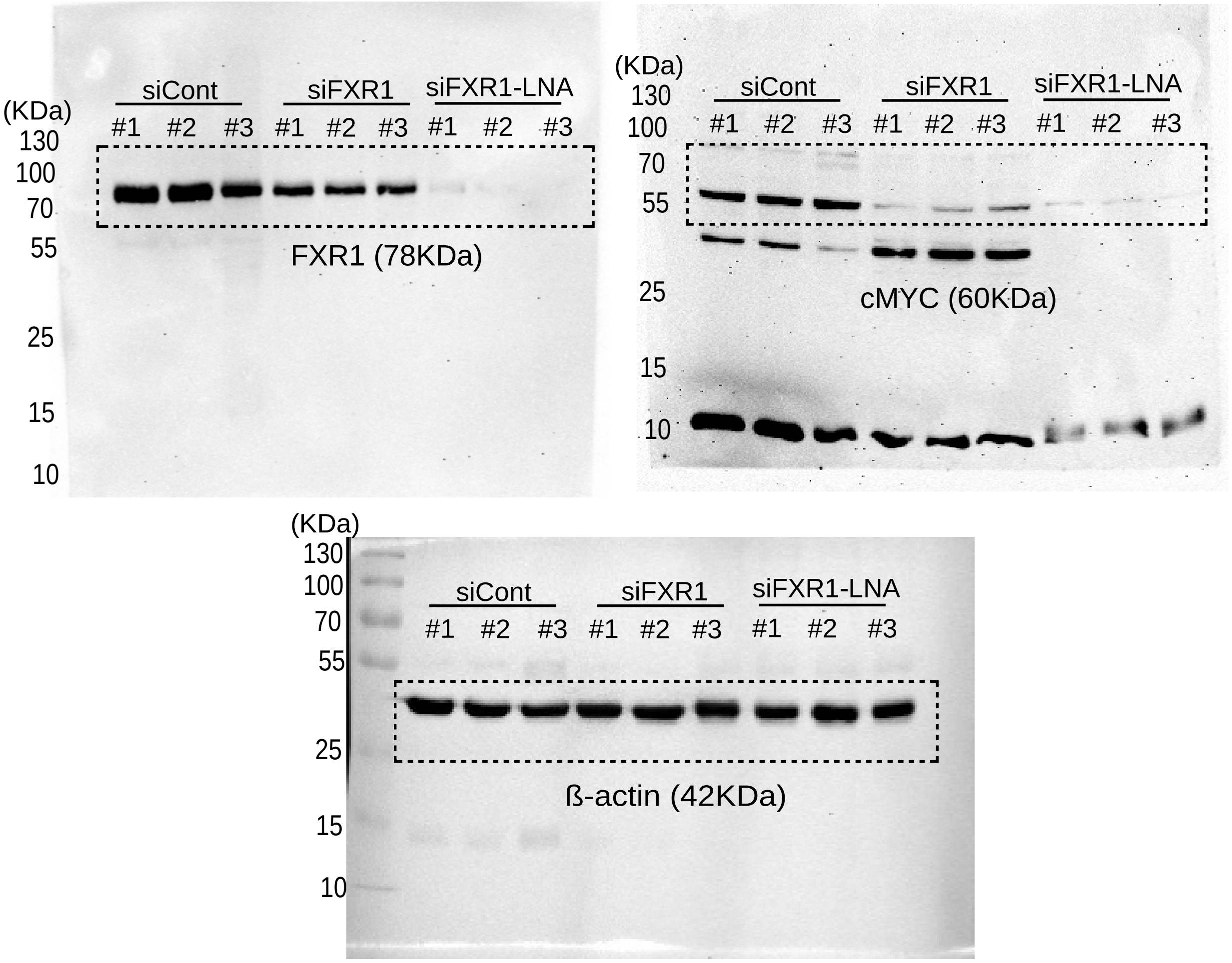

Supplement: Supplementary file 8 — Source Data file 1 [file 41467_2026_71468_MOESM8_ESM.pdf]
